# Construction of hyperbranched imprinted nanomaterials for selective adsorption of cadmium (II)

**DOI:** 10.55730/1300-0527.3664

**Published:** 2024-02-20

**Authors:** Sheng Nan HU, Xinyu ZHANG, Ming GUO, Shengchun WU, Wenjun LU

**Affiliations:** 1 Department of Chemistry and Materials Engineering, Zhejiang Agriculture & Forestry University, Hangzhou, China; 2 Department of Environmental and Resource Sciences, Zhejiang Agriculture & Forestry University, Hangzhou, China

**Keywords:** Halloysite, ion-imprinting, hyperbranched polymer, cadmium ion, specific adsorption

## Abstract

A hyperbranched ion-imprinted polymer (IIP) material containing multiple selective adsorption sites was synthesized using halloysite nanotubes, methyl acrylate, and ethylenediamine in the presence of a template ion [i.e. Cd (II) heavy metal]. The successful preparation of the Cd-IIP composition was confirmed by FT-IR, XRD, TEM, TGA, and elemental analysis. The polymers exhibited good adsorption of Cd (II) with a maximum adsorption capacity of 64.37 mg·g^−1^. The imprinting factor (α) for Cd (II) was 2.62 and the selection factor (β) was 1.78, indicating a specific adsorption of Cd (II) ion. The selection coefficients of Cd-IIP for Cd (II)/Pb (II), Cd (II)/Cu (II), Cd (II)/Ni (II), Cd (II)/Cr (III), and Cd (II)/Na (I) also indicated an excellent selectivity of the hyperbranched polymers for Cd (II) in the presence of competitive ions. The removal efficiency remained more than 75% after five cycles of desorption/adsorption. We envision that the HNTs based Cd-IIP has promising applications in the removal of Cd (II) from wastewater.

## 1. Introduction

Heavy metal ions are the major pollutants in wastewater [1,2]. The extensive heavy metal pollution of water resources caused by man-made or natural sources threatens access to clean drinking water globally [3]. In addition, exposure to low concentrations of these metal contaminants may cause cardiovascular, immune, neurological, and endocrine disorders, and cancers [4]. Many technologies have been developed to remove the metal ions from wastewater, such as ion exchange, filtration, solvent extraction, and sorption [5–11]. In particular, sorption is a facile and effective method due to its cost-effectiveness, versatility, and simplicity in removing trace-level of metal ions from aqueous systems [12].

Nanomaterial-based adsorbents have attracted paramount interest in the removal of metal ions due to the large surface areas, ease of modification, and multiple adsorption sites [13]. Nonetheless, a good separation of contaminants along with a remarkable reusability is still challenging for current nano-adsorbents. Furthermore, the residual nanomaterials in water may cause secondary pollution. Therefore, alternative adsorbents of excellent separation and regeneration properties are highly desirable.

The ion imprinting technique uses the target ion as a template for cross-linking polymerization, and the pores formed by the resulting polymer are specific, and the pore properties are related to the structure and size of the target ion [14–17]. For example, Zhang et al. [18] synthesized a novel N-doped carbon nanotube decorated with fish-scale molybdenum disulfide nanosheets (C-PPy@MoS_2_) by high-temperature hydrothermal reaction, which exhibited a maximum Pb (II) removal capacity of 381.87 mg·g^−1^. Guo et al. [19] designed and synthesized ZnNiCr layered double hydroxides for the removal of hexavalent chromium by microwave hydrothermal method with a maximum adsorption capacity of 28.2 mg·g^−1^. However, most ion-imprinted polymers still have the problems of low active site density, slow target-binding rates, low adsorption saturation, and poor specificity [20]. Specifically, the polymerization reaction causes the imprinted sites to be encapsulated, which is not conducive to adsorption [21]. Mehdinia et al. [22] prepared surface imprinted polymers of cadmium ions on amino-modified silane surfaces using graft polymerization reaction, a saturation adsorption capacity of 36.30 mg·g^−1^ for Cd (II). The grafted imprinted polymer increases the density and adsorption capacity of the active site and improves the defects of the polymerization reaction.

Halloysite nanotubes (HNTs) are nanomaterials discovered in recent years [23–27]. They feature in a hollow tubular structure, abundant interfacial hydroxyl groups, and high porosity. In addition, surface-modified HNTs are cost-effective, stable, and environmental-friendly carriers. Separately, the hyperbranched polymer forms a three-dimensional network structure and shows great potential for adsorption applications due to the multiple adsorption sites [28]. The combination of HNTs and hyperbranched polymers can achieve synergistic effect and enhance the removal efficiency of Cd (II). Heavy metal adsorbents satisfying the advantages of green, cheap and high adsorption capacity were prepared. Therefore, we hypothesized that the application of HNTs and hyperbranched polymers to the development of ion-imprinted materials could overcome the problems of low adsorption capacity and poor specificity faced by most current ion-imprinted materials.

In this study, we grafted the hyperbranched polymers onto the HNTs by surface imprinting polymerization [i.e. Cd (II)-imprinted polymers] for specific Cd (II) ion removal [29]. HNTs were used as the starting materials, and a dispersed synthesis route was used to introduce hyperbranched terminal amino groups on the surface [30]. The large number of imprinting sites were provided by the hyperbranched polymers, thus increasing the saturated adsorption capacity of target ions. Our heavy metal adsorbent is easy to prepare, inexpensive, highly specific, and has a large adsorption capacity compared with the traditional ion-imprinted polymers. In addition, the results showed that the polymer HNTs adsorbent retained 75% of Cd (II) removal after five desorption-adsorption cycles.

## 2. Experimental

### 2.1. Materials

Halloysites (HNTs) were purchased from Hebei Qingtai Mineral Products Co., Ltd. Ethyl ether, methanol, anhydrous ethylenediamine (EDA), cadmium nitrate, lead nitrate and chromium nitrate were purchased from Saen Chemical Technology Co., Ltd. Methyl acrylate (MA), 3-aminopropyltriethoxysilane (APTES), polyacrylamide (PAM), epichlorohydrin (ECH) and azobisisobutyronitrile (AIBN) were purchased from Sinopharm Chemical Reagent Co., Ltd.

### 2.2. Sample preparation

Synthesis of the terminal amino hyperbranched molecule NHNTs-3: To purify and decontaminate HNTs. We stirred 5 g of HNT in hydrochloric acid solution (35%) for 24 h, then washed 3 times with distilled water to obtain the acidified HNTs. The acidified HNTs were added to a mixture of toluene (25 mL) and APTES (7 mL) and reflow at 60 °C for 8 h under N_2_ protection. The mixture was then collected using filtration and washed with diethyl ether, ethanol and methanol 5 times for each solvent. The mixture was then dried at 60 °C to obtain amino-modified HNTs (AHNTs). The prepared AHNTs were added to ethanol (20 mL) followed by MA (10 mL). The mixture was stirred at 60 °C for 12 h, filtered and washed 5 times with methanol, then dried at 60 °C to obtain the intermediate MHNTs. Grinding yielded MHNTs that were grafted with MA. The prepared MHNTs and EDA (10 mL) were added to methanol (30 mL). The resulting mixture was stirred at 60 °C for 24 h, washed with methanol 5 times, then dried at 60 °C. Grinding yielded the product NHNTs-1. The process of grafting MA then EDA was repeated to obtain the terminal amino hyperbranched molecules on NHNTs-1 to give the terminal amino hyperbranched molecules NHNTs-2 and NHNTs-3 [31,32].

Synthesis of Halloysite-based ion-imprinted hyperbranched polymer (Cd-IIP): Cd (NO_3_)_2_ (20 mL, 0.1 M) was added to an Erlenmeyer flask with methanol (15 mL), NHNTs-3 (2 g) and PAM (1.5 g). After oscillating the mixture at 40 °C for 3 h, and then the cross-linking agent ECH (6.5 g) and initiator AIBN (0.15 g) were added. After uniform stirring, the mixture was heated and oscillated for 6 h at 50 °C in an N_2_ atmosphere. The resulting product added to a dilute hydrochloric acid solution (2 M), shaken at 50 °C in a shaker until Cd (II) could not be detected using atomic absorption spectrophotometer (AAS), they were then washed with methanol to neutral. The resulting Cd-IIP was then dried under vacuum at 60 °C. Nonimprinted polymer (NIP) adsorbents were fabricated using the same procedure without the addition of the template ions.

Fourier transform infrared spectroscopy (FTIR, IR Prestige-21, Japan) was used to characterize, this confirmed that the IIP was successfully prepared. Scanning electron microscope and energy spectrometer (SEM-EDS, JSM-7800F, Japan) were used for morphological analysis. Transmission electron microscope (TEM, Tecnai F30G2, United States) were used to analyze the apparent morphology of the sample. The Brunauer-Emmett-Teller (BET, NOVA, United States) method calculates the specific surface area.

The thermal properties of the samples were tested using a thermal analyzer (2090F3, Germany). The grafting rate was calculated from the thermogravimetric (TG) curves. Characterization of sample structure using nuclear magnetic resonance (NMR, Varian Inova-400, United States). The samples to be tested were mixed with deuterated chloroform reagent by sonication and dissolved and transferred to the NMR tube for testing. The data obtained were analyzed using MestReNova software.

### 2.3. Adsorption performance experiments

The adsorption and specific recognition of Cd (II) by the HNTs, Cd-IIP and NIP were investigated under different conditions. The amount of imprinted adsorption was calculated using the Lambert-Beer law and the Eq. 1.


(1) $$Q=\frac{(C_{0}-C_{e})\cdot V}{W}$$

where *Q* is the amount of adsorption, *W* is the mass of the polymer, *C_0_* is the initial concentration of the Cd (II), and *C_e_* is the concentration of the Cd (II) after adsorption equilibrium, *V* is the volume of the adsorbent.

Cd (NO_3_)_2_ solutions (20 mL, 100 mg·L^−1^) were added to Erlenmeyer flasks. The ion concentration in the supernatant was determined by AAS. The imprint factor (α) and the selection factor (β) were calculated using Eq. 2, and these were used to analyze the specificity of the different materials for Cd (II) ions.


(2) $$\alpha -Q_{IIP}/Q_{NIIP}\;;\;\;\;\beta =(Q_{IIP}-Q_{NIIP})/Q_{NIIP}$$

where *Q_IIP_* is the amount of adsorption of the substrate by the molecularly imprinted polymer; *Q_NIP_* is the amount of adsorption of the blank control substrate.


(3) $$K_{d}=\frac{C_{p}}{C_{s}}$$

(4) $$k=\frac{K_{d}(Cd(II))}{K_{d}(M)};\;\;\;k^{\prime }=\frac{k_{IIP}}{k_{NIIP}}$$

where *C_p_* is the concentration of the polymer bound substrate, and *C_s_* is the concentration of the substrate in the solution at adsorption equilibrium; *K_d_* (Cd (II)), *K_d_* (M) are the partition coefficients of Cd (II) and reference ions, respectively; *K_IIP_* and *K_NIP_* are the selectivity coefficients of Cd-IIP and NIP.

Pb (II), Cu (II), Ni (II), and Cr (III) were selected competitors for Cd (II) adsorption. Solutions containing Cd (II) ions (100 mg·L^−1^) and the competitor ions were prepared mixed solutions (100 mg·L^−1^). HNTs, Cd-IIP or NIP (20 mg) were added to the mixed solutions (20 mL) and shaken at 25 °C for 180 min. The concentration of metal ions in the solution was determined by AAS. The partition coefficient of NIP for each ion was calculated according to Eq. 3. Eq. 4 was used to calculate the selectivity coefficient (*k*) and the relative selectivity coefficient (*k*’).

## 3. Results and discussion

**Figure 1:**
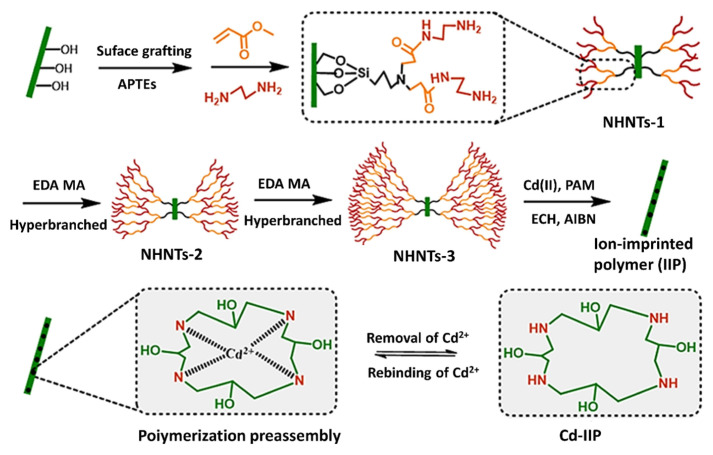
The synthetic route of hyperbranched ion-imprinted polymer (Cd-IIP) and 3rd generation amino-hyper hybridization.

Cd-IIP (Hyperbranched ion-imprinted polymers based on HNTs), NHNTs-1 (1st generation), NHNTs-2 (2nd generation) and NHNTs-3 (3rd generation) were prepared according to the reaction route (Figure 1). The silane coupling agent APTES (i.e. HNTs with silanol structures after APTEs catalysis) was chemically bonded to the surface of the HNTs to prepare the amino functionalized AHNTs. These amine groups underwent Michael addition to the double bond in MA and yielded the MA-modified MHNTs. The use of EDA allowed two amino groups to be grafted on MHNTs by amidation reaction and produced the NHNTs-1, which was sequentially reacted with MA and EDA to prepare the hyperbranched NHNTs-2 and NHNTs-3 structures. Then, by the surface ion-imprinting technique, using PAM as the functional monomer, Cd (II) as the template ion, and NHNTs-3 as the imprinted substrate, a complex was formed. The crosslinking agent ECH was added to the complex and prepared by crosslinking and polymerization with PAM in the presence of an initiator AIBN to obtain the ion-imprinted polymer (IIP). Finally, the template was eluted under acidic conditions and the hyperbranched ion-imprinted polymer with specific selective recognition to Cd (II) (Cd-IIP) was obtained.

### 3.1. Structural characterization of ion-imprinted polymer Cd-IIP

**Figure 2:**
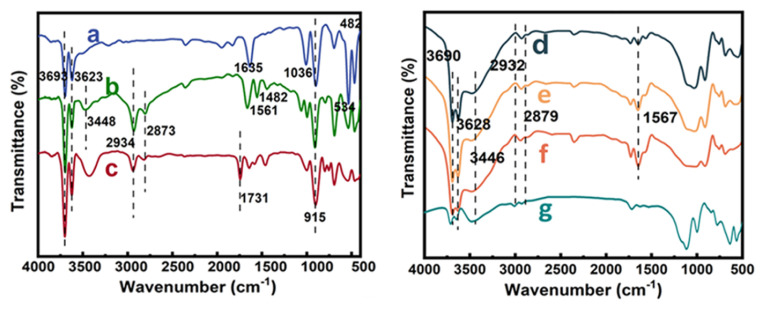
FT-IR spectra of HNTs (a), AHNTs (b), MHNTs (c), NHNTs-1(d), NHNTs-2 (e), NHNTs-3 (f), and Cd-IIP (g).

The results of FTIR spectra analysis show that the characteristic peaks of Al-O stretching vibrations (from the inner surface of the HNTs) were observed at 3623 and 3693 cm^−1^ (Figure 2a), while the bending vibrations from an intermediate layer of water on the HNTs were observed at 1635 cm^−1^. The peak at 915 cm^−1^ denotes the O-H bending vibration on the HNTs surface, and characteristic peaks of bending vibrations from Si-O, Al-O-Si and Si-O-Si were observed at 1036, 534, and 482 cm^−1^, respectively. The AHNTs retained the original absorption peaks that were observed from the HNTs (Figure 2b). At the same time, the N-H stretching vibration peak appeared at 3448 and 1561 cm^−1^, and C-H vibration peak appeared at 2934, 2873, and 1482 cm^−1^ [33,34].

The FTIR spectrum of the MHNTs exhibited a peak (1731 cm^−1^) from a carbonyl group, implying that the grafting of EDA was also successful (Figure 2c). The presence of N-H stretching and bending vibrations and the increased peak area of NHNTs-1 (Figure 2d), NHNTs-2 (Figure 2e), and NHNTs-3 (Figure 2f) indicate a higher generation of amination. Cd-IIP (Figure 2g) exhibited strong hydroxyl peak at 3450 and 1636 cm^−1^ compared to the FTIR spectrum of NHNTs-3 (Figure 2f), indicating that a ring-opening reaction occurred on the cross-linking agent epichlorohydrin. This indicates that the cross-linker was involved in the synthesis and the Cd-IIP, which laterally proves that Cd-IIP was successfully prepared. The peaks at 1084 and 542 cm^−1^ were characteristics of stretching vibration of Cd (II)-N, which confirmed that Cd (II) was successfully doped to the active sites. All the above analyses confirmed that the Cd-IIP was successfully synthesized.

**Figure 3:**
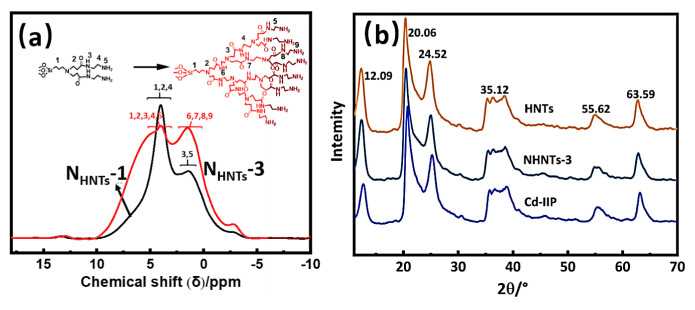
^1^H NMR spectrum of NHNTs-1 and NHNTs-3 (a), XRD diagram of HNTs, NHNTs-3, and Cd-IIP (b).

The results of ^1^H NMR spectrum analysis show that NHNTs-1 is the first generation of hyperbranched polymers with a simple molecular structure (Figure 3a). The resonance peaks of the -CH_2_- groups on the main chains of NHNTs-1 and NHNTs-3 were observed at δ = 4.0 ppm and δ = 4.1 ppm, respectively. In the main chains of NHNTs-1 and NHNTs-3, characteristic peaks of -NH_2_ were observed at δ = 1.4 ppm and δ = 1.6 ppm [35]. The integration region of NHNTs-3 widened, and the chemical shift is changed. This is probably due to the growing number of amino groups as the number of hyperbranched generations increases and the absorption band becomes wider. The peaks at δ = 4.1 ppm and δ = 1.6 ppm in the spectra are broad, with a slight change in the peak height. This is mainly due to the increasing degree of terminal amino substitution.

X-ray diffraction (XRD) analysis. The standard card of HNTs showed significant diffraction peaks at (NO.29-1487) at 2θ of 12.09°, 20.06°, 24.52°, 35.12°, 55.62°, and 63.59°. The characteristic diffraction peaks belonging to HNTs nanotubes are still present in the X-ray diffraction spectra of the structurally modified HNTs, NHNTs-3, and Cd-IIP (Figure 3b), but the magnitude of the diffraction peak intensity has changed, which indicating that the basic structure is not destroyed. The hyperbranched macromolecules on the surface modification of nanotubes existed in the amorphous form, which did not significantly affect the overall crystal structure of the products.

**Figure 4:**
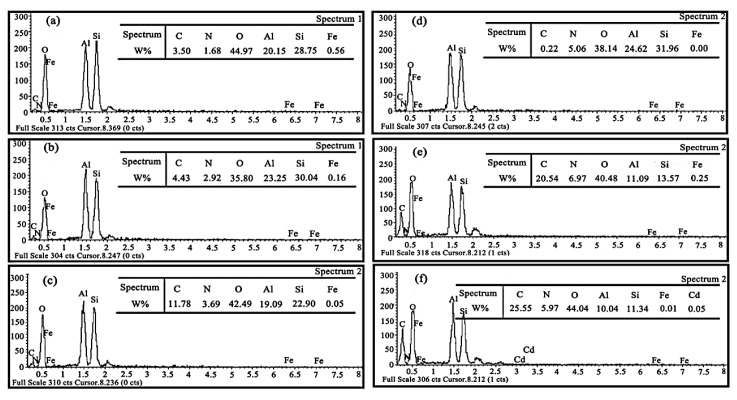
The EDS of HNTs (a), AHNTs (b), NHNTs-1 (c), NHNTs-2 (d), NHNTs-3(e), and Cd-IIP(f).

The EDS spectroscopy of HNTs and AHNTs shows (Figures 4a and 4b) that the nitrogen content increased from that the nitrogen content increased from 1.68% to 2.92% after the silane coupling process, while the oxygen content decreased from 44.97% to 33.80%, indicating that the APTES successfully reacted with the HNTs. As can be seen from Figures 4c–4e, the elemental N contents were 3.69%, 5.06%, and 6.97%, respectively, with a significant increasing trend of N content. The successful modification of supramolecular groups containing amino terminals on NHTs was confirmed. The carbon content increased from 20.54% to 25.55%, and the oxygen content increased from 40.48% to 44.04% (Figures 4e and 4f), confirming that the Cd-IIP was successfully prepared. The presence of traces of Cd in the EDS of Cd-IIP (Figure 4f) is caused by traces of Cd(II) ions remaining after the elution process.

**Figure 5:**
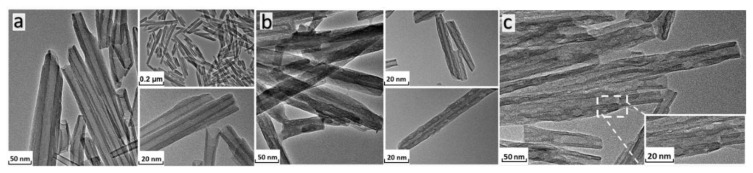
The TEM of HNTs (a), Cd-IIP (b), and NIP (c).

The results of TEM analysis show that the HNTs are hollow and transparent cylindrical nanotubes (Figure 5a). They are open at both ends, double-layered, and have a smooth surface. Cd-IIP (Figure 5b) and NIP (Figure 5c) had a bilayer and tubular morphology, which was significantly rougher than that of the HNTs. Although the surface of the NIP is irregular and flat, the surface of the Cd-IIP is rough, uneven, and porous. A comparison of the TEM images shows that the surface of NIP is rough and uneven with a loose and porous surface. This may be caused by the imprinted holes that remained after the template ions were eluted, which indicates that the target product was obtained.

The surface areas of HNTs, Cd-IIP and NIP were tested according to BET analysis by nitrogen adsorption, and the tested surface areas were 37.69, 76.96 and 42.1 m^2^·g^−1^, respectively [36]. It is clear from Table 1 that the total specific surface area and pore capacity of HNTs increased after modification, which indicates that the material preparation is consistent with the expectation [37]. It is that the specific surface area and pore capacity of NIP are smaller than those of Cd-IIP, but the average pore size of NIP is larger, which explains the formation of imprinted cavities of Cd-IIP [38].

**Figure 6:**
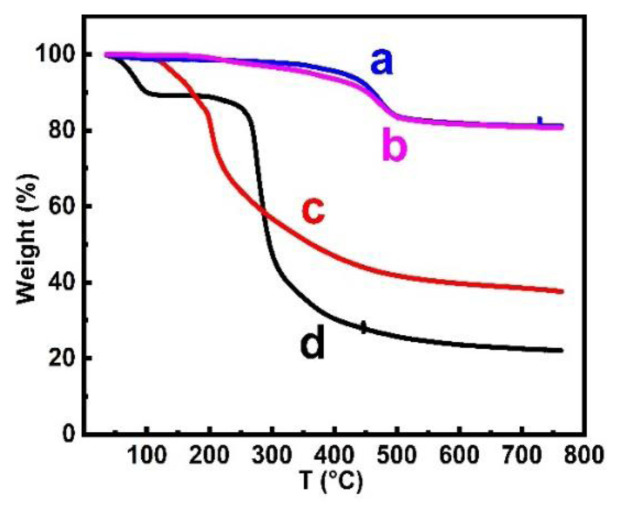
TG diagram of HNTs (a), AHNTs (b), NHNTs-3 (c), and Cd-IIP (d).

TGA plots of HNTs, AHNTs, NHNTs-3 and Cd-IIP showed (Figure 6) that all materials exhibited weight loss in the range of 0–150 °C, mainly due to the loss of adsorbed water. HNTs showed no significant weight loss at 700 °C, indicating that the thermal stability of the support is good. However, NHNTs-3 showed weight loss from 100–600 °C, which may be caused by the dehydrogenation of Al-OH. The weight loss of Cd-IIP from 250–600 °C was greater than that of NHNTs-3. This may be due to the increase in the content of pyrolytic components after the formation of imprinted polymers. The surface grafting of HNTs was successful by calculating the grafting rate (Table 2) by TG analysis.

### 3.2. Adsorption of Cd (II) ions by Cd-IIP

**Figure 7:**
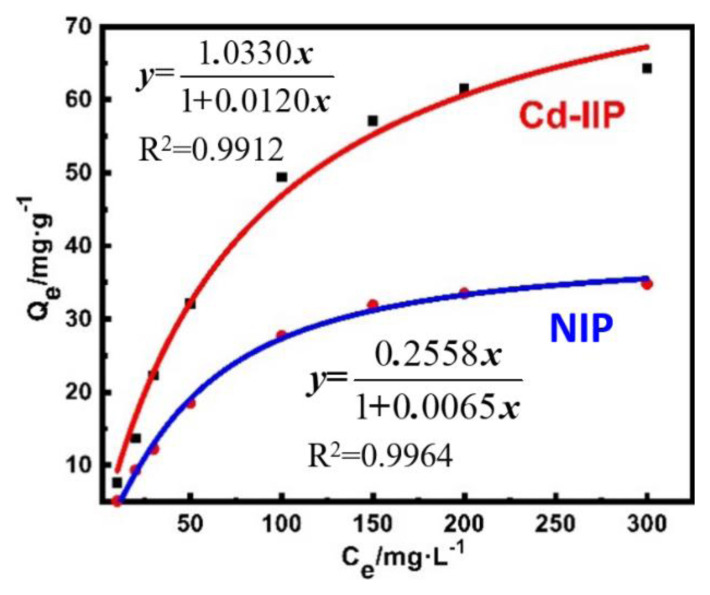
Isotherm adsorption curve of Cd-IIP and NIP.

The amount of Cd (II) that was adsorbed by Cd-IIP and NIP was calculated, and the adsorption isotherm was plotted (Figure 7). The amount of Cd (II) adsorbed by Cd-IIP and NIP increased with increasing substrate concentration. The saturated adsorption capacities of Cd-IIP and NIP are 64.37 and 34.8 mg·g^−1^, respectively. The isothermal adsorption equations of the two materials showed a good linear correlation.

**Figure 8:**
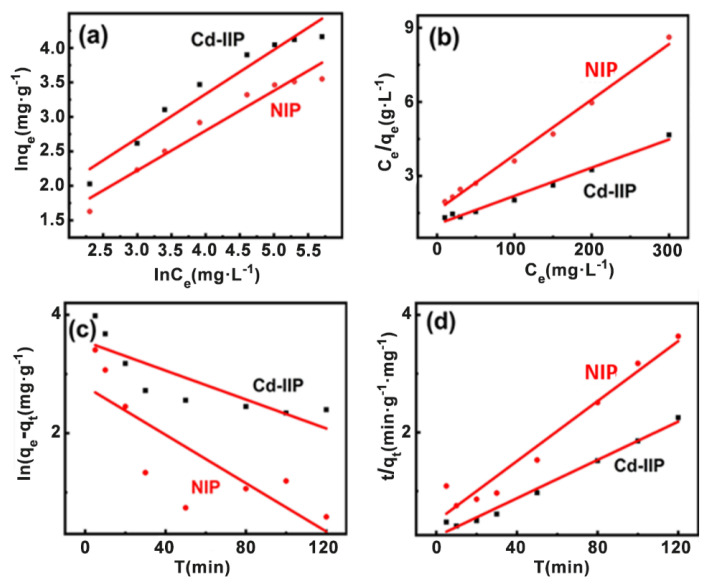
Freundlich isotherm adsorption (a), and Langmuir isotherm adsorption (b) model fitting curve, Quasi-first-order (c), and Quasi-second-order (d) dynamics curve.

Freundlich and Langmuir adsorption isotherm models (Eqs 5 and 6) were used to fit the data, and the kinetic adsorption curves (Figures 8a and 8b) were obtained [39,40].


(5) $${Inq}_{e}={lnK}_{F}+\frac{1}{\text{n}}{lnC}_{e}^{\prime }$$

(6) $$\frac{C_{e}^{\prime }}{q_{e}}=\frac{C_{e}^{\prime }}{q_{max}}+\frac{1}{q_{max}K_{L}}$$

where *C_e_’* is the ion concentration at equilibrium, *q_e_* and *q_max_* are the adsorption capacity at equilibrium and saturation, respectively, *K_F_* and *K_L_* are the adsorption constants of Freundlich and Langmuir, respectively.

The linear fitting results using the Langmuir model indicated that the maximum adsorption capacities of the two are 62.6 and 33.1 mg·g^−1^, respectively (Table S1), which was more similar to the actual adsorption saturation capacity. Additionally, the R^2^ values of the linear correlations using the Langmuir model are greater than 0.98, which is higher than those obtained by the Freundlich model. Therefore, the Langmuir adsorption isotherm model is more appropriate for modeling the thermodynamic adsorption behavior of Cd-IIP. This indicates that the distribution of active sites is more uniform, the adsorbent is a single molecule adsorption. The maximum adsorption capacity of Cd-IIP for Cd (II) shows that the ion-imprinted polymer had a strong recognition ability for the substrate.

**Figure 9:**
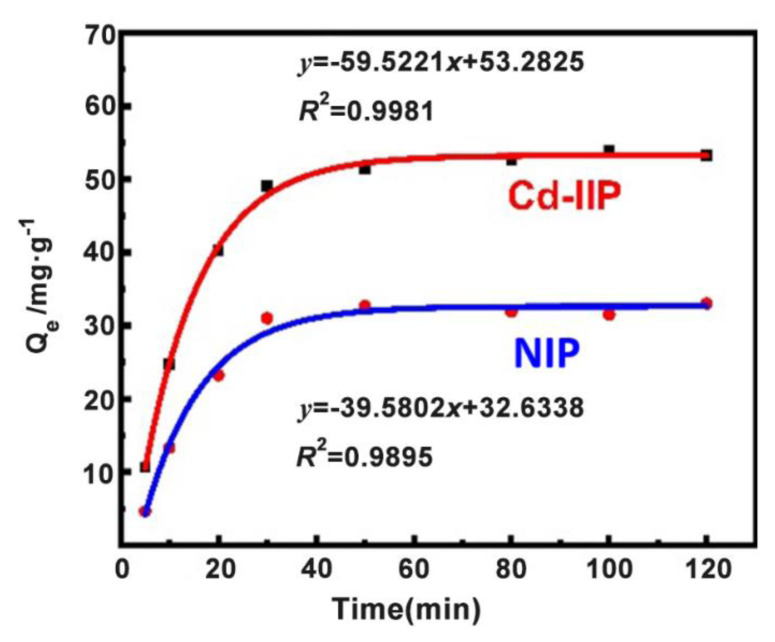
Adsorption kinetic curve of Cd-IIP and NIP.

Quasi-first-order and quasi-second-order kinetic models (Figures 8c and 8d) were used to fit the plots. Kinetic adsorption curves for the samples (Figure 9) were plotted.

First order dynamics


(7) $$ln\left(q_{e}-q_{t}\right)=lnq_{e}-K_{1}$$

Secondary dynamics


(8) $$\frac{t}{q_{t}}=\frac{1}{K_{2}q_{e}^{2}}+\frac{t}{q_{e}}$$

where *t* is the adsorption time; *q_e_* and *q_t_* are the adsorption amount of the adsorbed material to heavy metals when the adsorption reaches equilibrium; respectively, and *K_1_* and *K_2_* are the first and second adsorption rate constants.

The maximum saturation sorption amounts for Cd-IIP and NIP were 54.26 and 33.81 mg·g^−1^, respectively, with Cd-IIP far exceeding NIP. The results of the linear fit of the quasi-primary adsorption kinetic model gave a maximum adsorption of 34.75 and 16.29 mg·g^−1^ respectively, while the results of the linear fit of the quasi-secondary adsorption kinetic model gave 56.31 and 37.15 mg·g^−1^ respectively. It is noticeable that the equilibrium sorption amounts from the linear fit of the quasi-secondary sorption kinetic model are closer to the measured values and better reflect the kinetic behavior of the material for Cd (II) sorption.

The adsorption capacity of Cd-IIP is greater than that of NIP and HNTs, this is because in Cd-IIP, there are not only binding sites with template molecules, but also imprinted holes that are consistent with the stereoscopic structure of Cd (II). In NIP and HNTs, there are no imprinted holes that are complementary to Cd (II), and there are no sites that have specific adsorption capacity with Cd (II).

### 3.3. Adsorption selectivity of IIP

**Figure 10:**
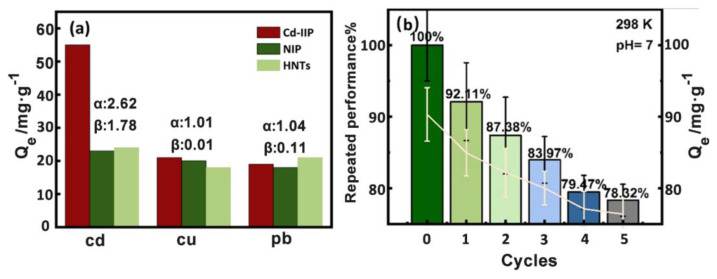
Selective adsorption effect of Cd-IIP, NIP, and HNTs on ions (a), performance of the ion-imprinted polymer Cd-IIP after 5 repeated adsorptions (b).

The adsorption selectivity results can be seen (Figure 10a) in that the adsorption capacity of Cd-IIP is greater than that of NIP and HNTs. The blotting factor α and the selection factor β were calculated by Eq. 3. The Cd-IIP has an imprinting factor (α) of 2.62 for Cd and a selection factor (β) of 1.78, there was no significant difference between Cu (II) and Pb (II) in α and β, indicating that Cd-IIP had good specificity for Cd (II).

The ion selection performance parameters of the Cd-IIP are given in Table S2. The Cd-IIP exhibited selection coefficients for Cd (II)/Pb (II), Cd (II)/Cu (II), Cd (II)/Ni (II), Cd (II)/Cr (III), Cd (II)/Na (I), Cd (II)/NO_3_^−^of 2.96, 1.80, 4.50, 4.57, 1.63, and 4.24 respectively, showing excellent selection performance for Cd(II). The larger the number, the better the selectivity of Cd-IIP for the ion. In the presence of competitive ions, the Cd-IIP exhibited excellent selectivity for Cd (II). In the presence of NO_3_^−^ and Na (I), Cd-IIP still showed excellent selectivity for Cd (II), however, due to the competition between cations, the selectivity decreased when Na (I) was present.

The adsorption-desorption experiments were repeated five times, and the recovery rate was determined as shown in Figure 10b [41]. After five cycles, the removal rate remained above 75%, the concentration of Cd (II) was 0.125 mg·L^−1^,highlighting the good regeneration performance of the Cd-IIP. At present, it is stipulated in China that the maximum allowable concentration of Cd (II) in sewage solution should be less than 0.5 mg·L^−1^, and the maximum allowable concentration in domestic water is 0.005 mg·L^−1^[42]. Through adsorption experiments and data analysis, it can be known that imprinted polymers have higher saturated adsorption capacity, better selection and recognition performance, and recycling performance.

## 4. Conclusion

In this study, a hyperbranched ion-imprinted polymer (Cd-IIP) based on HNTs was well-designed and prepared via a surface-ion-imprinted polymerization method. The Cd-IIP is easily regenerated and reused, and it was used for the adsorption of Cd (II) from an aqueous solution. The results showed that the adsorption data fit well with the quasi-secondary kinetics and Langmuir isotherm model, and the maximum adsorption capacity was 64.37 mg·g^−1^. Previously, Solic et al. [43] applied oxidized multi-walled carbon nanotubes (oxMWCNTs) as an effective adsorbent for the removal of Cd (II) from aqueous medium, and the adsorption equilibrium was reached within 20 min, with a maximum adsorption saturation of 13.50 mg·g^−1^. Alregeb et al. [44] successfully synthesized hyper-branched polyester nanoparticles by interfacial polymerization, and the adsorption amount of Cd (II) was 38.9 mg·g^−1^ at 45 °C. Qi et al. [45] synthesized a novel Cd (II) ion-imprinted polymer by native polymerization, which showed a maximum adsorption saturation of 50.54 mg·g^−1^ of Cd (II) in pH = 7 solution. In contrast, our hyperbranched ion-imprinted polymeric adsorbent Cd-IIP synthesized from HNTs is highly promising for the adsorption of Cd (II) in aqueous solution. In addition, the imprinting factors of 2.62, 1.01, and 1.04, and the selectivity factors of 1.78, 0.01, and 0.11 were determined for the adsorption of Cd (II), Cu (II), and Pb (II) pairs by Cd-IIP, indicating that Cd-IIP exhibited better adsorption performance and specific recognition of Cd (II) pairs. The results proved that Cd-IIP could be a specific adsorbent for Cd (II) solution, this work opens a window to develop high-performance sorbents for the removal of heavy metal ions from wastewater.

## Supplementary material

**Table S1:** Isotherm adsorption model fitting parameters and parameters of adsorption dynamic model equation.

	Freundlich isotherm adsorption					Langmuir isotherm adsorption			
	
	q _e_ (mg·g ^−1^)	Equation	n	K _F_	*R* *^2^*	q _max_ (mg·g ^−1^)	Equation	*n*	*R* *^2^*
Cd-IIP	64.300	*y* = 0.6409 *x* + 0.7689	1.560	2.1570	0.9400	62.600	*y* = 0.0114 *x* + 1.0518	0.010	0.9820
NIP	34.800	*y* = 0.5778 *x* + 0.4904	1.730	1.6330	0.9470	33.100	*y* = 0.0224 *x* + 1.6101	0.013	0.9910

	Quasi-first-order dynamics				Quasi-secondary dynamics				
	
	Equation		*q* _e1_ (mg·g ^−1^)	*K* _1_	*R* *^2^*	Equation	*q _e_* _2_ (mg·g ^−1^)	*K* _2_	*R* *^2^*

Cd-IIP	*y* = −0.0120 *x*+3.5480		34.7500	0.0120	0.6645	*y* = 0.0160 *x*+0.2260	56.3100	0.0012	0.9830
NIP	*y* = −0.0200 *x*+2.7910		16.2900	0.0200	0.6076	*y* = 0.0250 *x*+0.4890	37.1500	0.0013	0.9480

**Table S2:** Cd-IIP ion selection performance parameters.

Metal ion	q (mg·g ^−1^)	K _d_ (L·g ^−1^)	K _IIP_	Metal ion	q (mg·g ^−1^)	K _d_ (L·g ^−1^)	K _IIP_
Cd (II) Pb (II)	37.220 15.040	0.330 0.111	2.961	Cd (II) Cr (III)	26.670 6.7800	0.216 0.047	4.568
Cd (II) Cu (II)	35.920 22.320	0.315 0.175	1.801	Cd (II) Na ^+^	22.08 20.12	0.104 0.092	1.6318
Cd (II) Ni (II)	29.970 7.880	0.250 0.055	4.503	Cd (II) NO _3_ ^−^	31.89 13.73	0.286 0.108	4.235

## Figures and Tables

**Table 1:** Physical properties of hyperbranched ion-imprinted polymers.

Samples	S_BET_ (m^2^·g^−1^)	Average pore size (nm)	Pore volume (cm^3^·g^−1^)
HNTs	37.69	20.41	0.28
Cd-IIP	76.96	11.28	0.43
NIP	42.10	22.68	0.31

**Table 2:** The grafting rate of AHNTs, NHNTs-3, and Cd-IIP.

Samples	Grafting ratio /%	

	Experimental value	Theoretical value
AHNTs	5	15
NHNTs-3	143	163
Cd-IIP	406	422
